# Non-monotonous dose response function of the termination of spiral wave chaos

**DOI:** 10.1038/s41598-022-16068-8

**Published:** 2022-07-14

**Authors:** Thomas Lilienkamp, Ulrich Parlitz, Stefan Luther

**Affiliations:** 1 Max Planck Institute for Dynamics and Self-Organization, Göttingen, 37077 Germany; 2 German Center for Cardiovascular Research (DZHK), Partner Site Göttingen, Göttingen, 37075 Germany; 3 Institute for the Dynamics of Complex Systems, Georg-August-Universität Göttingen, 37077 Göttingen, Germany; 4 University Medical Center Goettingen, Institute of Pharmacology and Toxicology, 37075 Göttingen, Germany

**Keywords:** Biophysics, Computational biophysics, Nonlinear phenomena, Cardiology

## Abstract

The conventional termination technique of life threatening cardiac arrhythmia like ventricular fibrillation is the application of a high-energy electrical defibrillation shock, coming along with severe side-effects. In order to improve the current treatment reducing these side-effects, the application of pulse sequences of lower energy instead of a single high-energy pulse are promising candidates. In this study, we show that in numerical simulations the dose-response function of pulse sequences applied to two-dimensional spiral wave chaos is not necessarily monotonously increasing, but exhibits a non-trivial frequency dependence. This insight into crucial phenomena appearing during termination attempts provides a deeper understanding of the governing termination mechanisms in general, and therefore may open up the path towards an efficient termination of cardiac arrhythmia in the future.

## Introduction

Life-threatening cardiac arrhythmia like ventricular fibrillation (VF) are the major cause of morbidity and mortality with sudden cardiac death taking several hundreds of thousands of lives per year^1^. The complex and chaotic dynamics of the underlying electrical excitation patterns in the heart muscle is still not fully understood. However, it is known that spiral or scroll waves are the governing objects which drive the spatio-temporal dynamics during arrhythmia^2–5^. Today, the conventional treatment of VF consists of the application of an electrical defibrillation shock, aiming to restore sinus rhythm. Due to the significant electrical current induced in the cardiac muscle, this treatment comes along with severe side-effects, including additional tissue damage^6,7^ and post-traumatic stress^8^. Efforts to reduce these side effects have led to further improvements in cardiac defibrillation therapy and reduction of energy requirements. So far, the waveform of the single conventional defibrillation pulse has been studied and optimized, for instance, based on the capacitance of the defibrillation system^9^ or by comparison of monophasic vs. biphasic pulses^10^.

However, the physical principles underlying conventional cardiac defibrillation limit the maximum energy reduction that can be achieved. Therefore, alternative low-energy defibrillation techniques have been introduced, which have demonstrated their significant energy reduction potential in pre-clinical experiments and first clinical trials. In contrast to conventional defibrillation, in which a single electrical shock terminates all cardiac activity at once, most low-energy defibrillation approaches use sequences of weak electrical field pulses.

These electrical pulses interact with endogenous heterogeneities in the electrical conductivity of cardiac tissue, resulting in the formation of virtual electrodes (VE)^11,12^. The local VE-induced tissue depolarization can be used to perturb the excitation waves and eventually terminate fibrillation. The pacing sequences are optimized to achieve a specific purpose.

Currently, different approaches follow the aim to lower these side-effects, while maintaining a high reliability regarding the successful termination of occurring arrhythmia. For example, among the investigation of efficient electrical field configuration^13^, sequences of pulses of lower amplitude were used instead of a single defibrillation pulse, and their potential for the termination of arrhythmia has been studied^14–16^. A comprehensive numerical study of the dependence of termination efficacy of pacing sequences on frequency and number and amplitude of pulses has been conducted by Buran et al.^17^.

In many of these studies, the temporal distance between consecutive pulses is chosen based on the governing time scale of the system, which, for example, can be estimated in terms of the cycle length (temporal distance between consecutive depolarizations), a dominant frequency (largest amplitude of Fourier spectrum of, e.g. the electrocardiogram), or the time scale of spiral wave rotations (i.e., regarding the underlying electrical wave dynamics). The mechanisms and efficacy of spiral wave termination using pulse sequences with higher or lower pacing frequencies relative to the dominant frequency of fibrillation (i.e., overdrive or underdrive pacing) has been investigated in several studies^18–21^.

The relation between the applied electrical current (dose) and the successful defibrillation probability (response) is described by the dose-response function. For cardiac defibrillation using a single pulse, the dose-response function has a monotonously increasing sigmoidal shape^22^. A detailed characterization of the dose-response function requires a sufficiently large number of measurements, which is often impractical in experimental or clinical studies. Therefore, algorithms have been developed to estimate the defibrillation threshold from a limited number of defibrillation attempts^23^. These estimators assume prior knowledge of the form of the dose-response curve, i.e., a monotonous increase of success rate with shock energy. Also regarding the development of new termination concepts, the dose-response function plays a key role in many experimental and numerical studies^24^, as novel defibrillation approaches aim to provide a high termination rate, while keeping the pulse energy as low as possible.

Here we show that for multi-site pacing protocols the dose-response can be non-monotonic with high success rates at low pulse energies. This observation may have significant implications for the development and optimization of low-energy defibrillation. We demonstrate this effect in numerical simulations of 2D excitable media and show the robustness of this observation.

In the following section, we introduce the cardiac cell models used in this study and describe how termination rates were determined. Then, we compare the dose-response of (conventional) single pulse defibrillation with periodic sequences of 5, 10, and 20 pulses at different pacing frequencies. We also show that periodic multi-site pacing protocols result in complex dynamics of rotors or phase singularities present in the system, due to the transient creation and annihilation processes of spiral waves. We discuss these results and their potential implications for the non-linear dose-response in the last section.

## Methodology

All simulations of this study have been performed on two-dimensional rectangular simulation domains. More technical details about how the simulations have been performed can be found in the method section.

### Overview of investigated cardiac cell models

**Figure 1:**
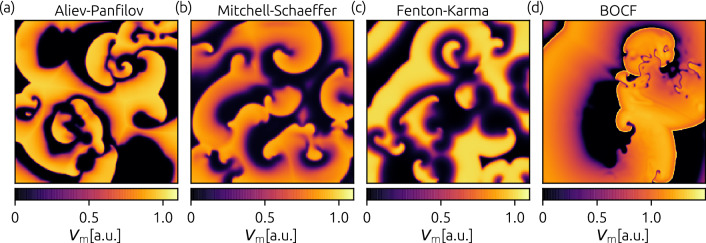
Exemplary snapshots of the membrane voltage $\mathrm V_m$ during episodes of spiral wave chaos are shown for the investigated cell models: the Aliev-Panfilov model AP (**a**), the Mitchell–Schaeffer model MS (**b**), the three-variable Fenton-Karma model FK (**c**), and the Bueno-Orovio-Cherry-Fenton model BOCF (**d**), respectively.

In order to cover a broad range of existent cardiac cell models and situations, we investigate in the following four different cell models, which describe the electrical action potential dynamics of cardiomyocytes: the Aliev-Panfilov model^25^ (from now on denoted as AP), the Mitchell–Schaeffer model^26^ with the adaption described by Álvarez et al.^27^ (MS), the Fenton-Karma model^28^ (FK), and the Bueno-Orovio-Cherry-Fenton model^29^ (BOCF). Details about the cardiac models and parameters are given in the method section. In Fig. 1, exemplary snapshots of the membrane potential $\mathrm V_m$ are shown for each model, respectively.

### Initializing chaotic states

For each cell model, we simulated ten independent episodes of chaotic spiral wave dynamics. In a second step, we performed termination attempts (consisting of different pacing sequences) to these initial conditions in order to compute associated termination rates. Initial conditions were created by applying spatial randomized perturbations to the dynamical variables of a state already showing spiral wave dynamics. After the application of the perturbation, we let the system evolve for a temporal period of $5\,\hbox {s}$, such that differently perturbed states are independent from each other. The states after the transient evolution time were then used as the actual initial conditions. Also, we evolved these initial conditions for another $5\,\hbox {s}$, in order to exclude self-termination of the chaotic dynamics^30–32^, which would corrupt later investigations regarding termination attempts.

### Implementation of termination attempts

**Figure 2:**
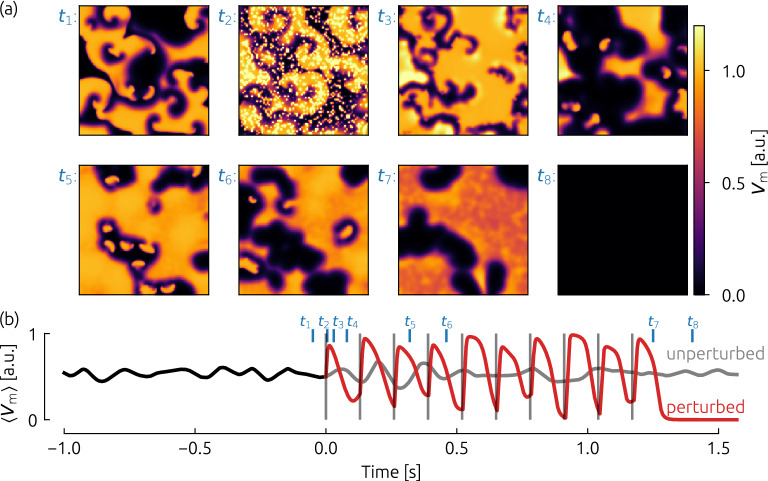
Exemplary (successful) termination attempt based on the Fenton-Karma model (FK) by a sequence of ten pulses. The frequency of the pulses in this example is equal to the dominant frequency $f_\mathrm{dom}$, and the amplitude of the pulses is $E=0.5$ a.u. In (**a**), eight snapshots of the membrane potential are shown just before the pacing protocol starts ($t_1$), during the pacing ($t_2$ - $t_6$) and after the final pulse ($t_7$ and $t_8$). The spatially averaged membrane potential $\langle V_\mathrm{m} \rangle$ (as a simple estimate for a pseudo ECG) is depicted in subplot (**b**), where pulses are marked with gray vertical lines and time instances which refer to the snapshots in (**a**) are marked in blue. From $t=0$ s, the red curve shows the case where the pacing protocol was applied, whereas the gray curve depicts the unperturbed case.

After creating initial conditions which exhibit chaotic spiral wave dynamics, we applied different termination schemes, consisting of sequences of electrical stimuli, and computed the associated success rates in terms of the ability to terminate the chaotic dynamics. We tested a single pulse protocol (as it is used for conventional defibrillation), as well as sequences of five, ten, and twenty pulses. An exemplary episode of a ten pulse sequence (FK model) is shown in Fig. 2.

We modeled the application of stimuli motivated by the concept of virtual electrodes, which is the underlying mechanism for electric field-induced wave emission and defibrillation^33^. As it was shown in experimental and numerical studies, when applying an electrical field to cardiac tissue, new excitation waves can be created at locations of varying electrical conductivity, or heterogeneities^15,17^, also called virtual electrodes. In our study, we implemented a simple model for virtual electrodes, where new excitation waves at specific locations are not generated by heterogeneities of the conductivity, but by local current injections. Therefore, before any termination attempt a certain number of locations (from now on called artificial virtual electrodes (AVE)) are chosen randomly, such that the relative fraction $A_\mathrm{cov}$ of the whole simulation domain is covered. During a pulse sequence, these locations were fixed. For significantly lower numbers of $A_\mathrm{cov}$, the chaotic dynamics can not be terminated with the considered pulse sequences used (significantly increasing the locally injected current would destabilize the local cell model). Too high numbers of $A_\mathrm{cov}$ would result in leaving the low-energy regime, that is characterized by a relatively low number of excitation sites. This fraction, as well as the absolute number of perturbation sites $N_\mathrm{ave}$, and the respective spatial size $S_\mathrm{pert}$ of single AVEs is given for each cardiac cell model in Table 1. Also, for characterizing the temporal and spatial properties of the dynamics, the dominant frequency $f_\mathrm{dom}$ and the average number of spiral waves $N_\mathrm{sp}$ is given. The latter one, was determined based on an episode with the temporal length of $10\,\hbox {s}$ of unperturbed spatio-temporal chaotic wave dynamics (details about the procedure can be found in^34^]).

**Table 1:** This table lists for all investigated cell models the dominant frequency $f_\mathrm{dom}$, the average number of spiral waves $\langle N_\mathrm{sp} \rangle$, the average transient lifetime $\langle T_\mathrm{trans} \rangle$, the spatial size of single AVEs $S_\mathrm{pert}$, the relative fraction of the simulation domain covered with AVEs $A_\mathrm{cov}$, the absolute number of AVEs $N_\mathrm{ave}$, and the temporal duration of single stimuli $t_\mathrm{dur}$.

Model	$f_\mathrm{dom}$ [$\hbox {H}_\mathrm{z}$]	$\langle N_\mathrm{sp} \rangle$	$\langle T_\mathrm{trans} \rangle$ [s]	$S_\mathrm{pert}$ [$\hbox {mm}^2$]	$A_\mathrm{cov}$ [%]	$N_\mathrm{ave}$	$t_\mathrm{dur}$ [ms]
AF	22.52	19.4 ± 6.8	77.9	$0.5\, \times \, 0.5$	30	12000	2
MS	2.94	19.1 ± 4.0	$\gg$ 100	$0.8\, \times \, 0.8$	30	6750	2
FK	7.69	19.3 ± 4.3	5071	$2.0\, \times \, 2.0$	25	1406	2
BOCF	2.68	11.8 ± 5.1	64.5	$1.6\, \times \, 1.6$	25	20736	2

When a single stimulus is applied to the tissue, a local current is injected at each position of an AVE for a period of $t_\mathrm{dur}=$ $2\,\hbox {ms}$. The amplitude of the injected current is used as a maximal amplitude, and for each AVE, the individual current amplitude was chosen randomly out of the interval [0, amplitude]. With this approach, we take into account that in living cardiac tissue the intensity of the depolarization at different virtual electrodes varies, for example due to curvature of the shape of the heterogeneity^12^. In practice, this leads to the occurrence of sub-threshold and supra-threshold stimuli of the tissue, at different AVEs, respectively. In the second snapshot ($t_2$) in Fig. 2(a), the system is shown after the application of such a stimulus.

For determining the success rate of a specific pulse sequence, we performed twenty termination attempts for each of the ten initial conditions (each with a different distribution of AVEs), leading to in total $10 \times 20 = 200$ termination attempts. The success rate for this case is then measured by the fraction of successful attempts. A termination attempt was denoted as successful, if after a period of $3/ f_\mathrm{dom}$ the membrane potential $V_\mathrm{m}$ was below $V_\mathrm{m}^\mathrm{thresh} = 0.1$ a.u. on the whole simulation domain. Since self-termination of spiral wave dynamics has been investigated before^30–32^ we also computed the average transient lifetime for these systems (followed the procedure in^31^) and present the numbers in Table 1. Note that for the MS model, none of initially 1000 initial conditions terminated within $100\,\hbox {s}$. For each model, the average transient lifetime is much larger than the period of time we wait after the application of a pulse sequence ($T_\mathrm{wait} = 3/ f_\mathrm{dom}$). Hence, the influence of self-termination can be neglected.

## Results

In this section, we present the results regarding the termination of the four investigated cardiac cell models with sequences of 1, 5, 10, or 20 pulses.

### Frequency dependent success rate

As we discussed in the introduction, when applying a sequence of electrical defibrillation pulses to fibrillating hearts, the frequency of the applied pulses can play a key role. Therefore, regarding the dose-response function, the frequency of the pulse sequence becomes (in addition to the pulse amplitude) a second open parameter which impacts the success probability to terminate the arrhythmia. For a systematic investigation, we therefore computed the success rates for a broad range of pulse amplitudes and pulse frequencies. As we discussed earlier, the “dominant frequency” can be interpreted as the governing time scale of the system. Therefore, we investigated pulse frequencies close to $f_\mathrm{dom}$ in an interval of  $[0.5\, f_\mathrm{dom}, 1.5\, f_\mathrm{dom}]$. Details about how the dominant frequency is determined can be found in the method section.

**Figure 3:**
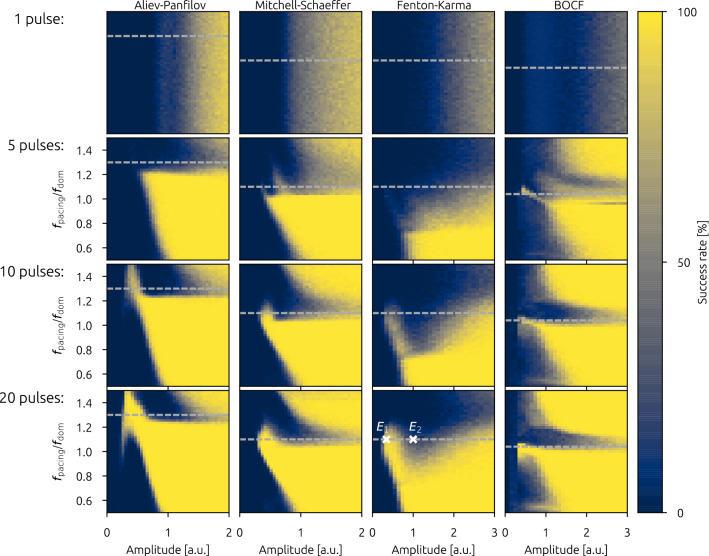
Overview of success rates depending on the pacing frequency. Each column depicts results for each investigated cardiac cell model, whereas the rows denote the number of pulses delivered, respectively (1 pulse, five pulses, ten pulses, and twenty pulses). The pacing frequency $f_\mathrm{pacing}$ is given in multiples of the dominant frequency $f_\mathrm{dom}$ which was determined for each cell model individually. Note that the frequency dependence for a single pulse is technically redundant, due to the lack of subsequent pulses. However, the data is still shown in order to demonstrate the magnitude of statistical fluctuations regarding the determination of the success rate. Horizontal lines at specific pacing frequencies denote dose-response curves, which are shown explicitly in Fig. 4. Two specific combinations of frequency and energies $E_1$, and $E_2$ (marked by white crosses) are discussed in more detail later.

In Fig. 3, a comprehensive presentation of the success rate (color coded) is given for each cardiac cell model (column-wise), respectively. The results for different numbers of pulses are presented in each row (single pulse, five pulses, ten pulses, and twenty pulses), and in each subplot, the success rate of the specific pulse sequence is given for different generalized energies whereas the pacing frequency $f_\mathrm{pacing}$ is given in multiples of the dominant frequency $f_\mathrm{dom}$.

As a clarification, in the subplots of Fig. 3 horizontal lines with a fixed pulse frequency could be interpreted as conventional dose-response functions. Also, technically the concept of a varying pulse frequency does not make sense in the case of a single pulse. Still, we presented the single pulse data in a similar form as the multi-pulse data (for the single pulse, the y-axis denotes different realizations of AVEs, only), in order to give an impression of the magnitude of statistical fluctuations of the success rate. In general, we can observe in all investigated cell models a non-trivial pattern of the success rate, depending on both, the pulse amplitude and the pacing frequency. In particular, with increasing pulse numbers, a protrusion of significant success rate is forming, heading towards lower energies and towards pacing frequencies higher than the dominant frequency. While this general pattern can be observed in each case, the detailed shape and width of the protrusion depends on the respective cell model investigated. However, for higher pulse numbers the size of the pattern (in terms of success rate, energy range and frequency range) is increasing.

**Figure 4:**
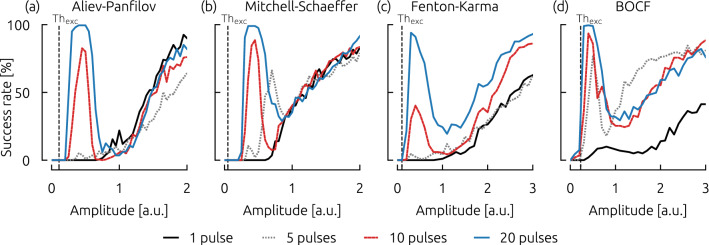
Dose-Response curves for selected pacing frequencies ((**a**) AP: $1.3 f_\mathrm{dom}$, (**b**) MS: $1.1 f_\mathrm{dom}$, (**c**) FK: $1.1 f_\mathrm{dom}$, (**d**) BOCF: $1.04 f_\mathrm{dom}$). The success rates of the selected pacing frequencies are marked as horizontal gray dashed lines in Fig. 3.

The observation of this complex dependence of the success rate on pulse amplitude and frequency can therefore lead to uncommon, non-sigmoid and non-monotonous dose response functions. For each model, exemplary dose response functions are shown in Fig. 4 for specific pacing frequencies ((a) AP: $1.3 f_\mathrm{dom}$, (b) MS: $1.1 f_\mathrm{dom}$, (c) FK: $1.1 f_\mathrm{dom}$, (d) BOCF: $1.04 f_\mathrm{dom}$) which are marked in Fig. 3 as horizontal gray dashed lines.

For dose-response functions with a fixed pulse frequency, we can observe local maxima, or peaks of significant success rates at relatively low pulse energies, where the success rate decreases afterwards, although the pulse energy is increased. We find, that the height of this peak (in terms of the success rate) and the width (in terms of energy range) becomes larger with increasing pulse numbers. Also, in some cases, the maximum success rate of the peak shifts with different pulse numbers (as for example in Fig. 4(b) MS: comparison of five pulses and ten pulses). Since we observe significant success rates at low pulse amplitudes, we also checked whether the termination mechanism is mainly based on sub- or suprathreshold termination. For this purpose, we determined the excitation threshold $\textrm{Th}_{\rm exc}$ for each cell model (by applying stimulations into resting tissue) and marked it by vertical black dashed lines in Fig. 4. Although in the case of the BOCF model, there is no clear separation between non-zero success rates and the excitation threshold, we suppose that the governing termination mechanism is based on supra-threshold termination.

Furthermore, we showed that the qualitative results remain robust if longer pulse durations are used. In Fig. S1 of the supplement dose-response functions are shown, similar to Fig. 4 but with pulse duration of $4\,\hbox {ms}$ instead of $2\,\hbox {ms}$. The increased pulse duration leads to more distinct maxima at low energies. However, varying pulse duration does not qualitatively change the main findings.

Regarding the efficient termination of chaotic spiral wave chaos (and the related application of terminating cardiac arrhythmia) it is noteworthy, that the local maxima represent areas with a significant success rate combined with the lowest pulse energies we found in the simulations. Therefore, the investigated regimes may be of high interest for the development and understanding of novel efficient termination strategies using pacing frequencies.

### Frequency dependent temporal evolution of NPS

For a robust exploitation of the frequency dependent occurrence of high success rates at low amplitudes towards an efficient termination of the chaotic spiral wave dynamics, it is necessary to obtain further insight into the underlying mechanism leading to the patterns discussed in Figs. 3 and  4. As a first empirical investigation, we therefore discuss the temporal evolution of the number of phase singularities (as an estimate for the number of spiral waves present in the system) exemplary for simulations of the FK model. For this purpose, the average number of phase singularities was tracked during the pacing process (twenty pulses) for 200 simulations with a fixed pulse frequency of $f_\mathrm{pacing} = 1.1 f_\mathrm{dom}$ and two different pulse energies $E_1$ and $E_2$. Details about how NPS were detected can be found in the method section. We chose these parameter combinations (marked as white crosses in Fig. 3) such that the dynamics of the “protrusion” can be studied at the peak, and also at the following local minima.

**Figure 5:**
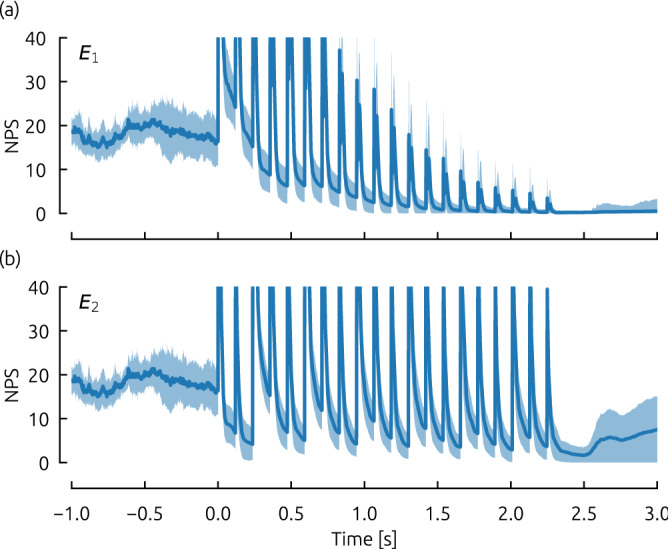
The temporal evolution of the number of phase singularities (NPS) as a measure for the number of spiral waves in the system during a ten pulse protocol. For the Fenton-Karma model (FK), two pulse amplitudes were selected ($E_1=0.3$ shown in (**a**) and $E_2 = 1.0$ shown in (**b**)) for a specific pacing frequency of $f_\mathrm{pacing} = 1.1 f_\mathrm{dom}$ (marked in Fig. 3 as white crosses). For these cases, the number of phase singularities was tracked over time for 200 simulations. After averaging over all simulations, the mean NPS is depicted in blue, whereas the standard deviation is marked in light blue.

In Fig. 5(a) and (b), the temporal evolution of the average NPS is shown for both energies $E_1$, and $E_2$, respectively as the blue curve. Light blue color indicates the standard deviation, based on 200 simulations analyzed. During the period prior to the pacing protocol (at $t=$ $0\,\hbox {s}$), the number of phase singularities fluctuates around values of $\approx 10-25$. With the beginning of the pacing sequence, the NPS increases significantly shortly after the application of each single stimulus. During this short period, the large number of phase singularities cannot be linked to the number of spiral waves directly, but is rather biased by the impact of the pacing process on the way the NPS are computed. Apart from these fluctuations, which appear for both pacing amplitudes, a significant difference between the evolution of NPS for $E_1$, and $E_2$ is that the baseline of NPS (ignoring the stimulus induced peaks) is converging to zero in the first case of $E_1$ (a), but is rather fluctuating in the case of $E_2 > E_1$. Therefore, after the pacing sequence has finished, cardiac tissue has (for most simulations) not been synchronized completely, and remaining spiral waves cause the perpetuation of spiral wave chaos.

## Discussion

In this study, we present a numerical investigation regarding the termination of two-dimensional spiral wave chaos with sequences of electrical pulses. In particular, we show how for different cardiac cell models, the success probability of spiral wave termination of a sequence of pulses depends in a complex way on the pulse amplitude and the pulse frequency. We found a non-trivial relation between these two parameters, leading to non-monotonous (and non-sigmoidal) dose response curves which exhibit a local maximum at relatively low pulse energies, followed by a local minimum at higher energies. This indicates, that in this frequency regime electrical synchronization (measured by the number of phase singularities) of cardiac tissue is more efficient for lower energies than for intermediate ones.

Although a clear relation between amplitude and frequency could not be derived so far, the regime where local maxima of high success rates and low energies appeared can be of high interest for future termination studies, since they provide the ability to efficiently terminating the chaotic dynamics with significantly lower pulse amplitudes in each cell model. Furthermore, although the detailed shape, width, and structure of the success rate patterns differed in the investigated cell models, the discussed regime appeared at frequencies slightly faster than the dominant frequency $f_\mathrm{dom}$, which in the literature is often denoted as “overdrive pacing”^19,20^. Therefore, the study presented and follow-up studies could provide insights into the underlying mechanisms of how the performance of such overdrive protocol can be further increased.

In general, this study provides a new insight into complex phenomena, which can occur during the synchronization and termination of excitable media and will hopefully provide new impetus regarding low energy termination of cardiac arrhythmia.

## Methods

### Cardiac cell models

More details about the investigated cardiac cell models are given in this section. The dynamics is determined by a system of reaction-diffusion equations Eqs. (1) and (2):

1 $$\begin{aligned} \frac{\partial V_\mathrm{m}}{\partial t}= & {} D \Delta V_\mathrm{m} - \mathbf {f}(V_\mathrm{m}, \mathbf {h})\, , \end{aligned}$$

2 $$\begin{aligned} \frac{\partial \mathbf {h}}{\partial t}= & {} \mathbf {g}(V_\mathrm{m}, \mathbf {h}) \, , \end{aligned}$$

where *D* is the diffusion constant. The exact form of the transmembrane currents $\mathbf {f}(V_\mathrm{m}, \mathbf {h})$ (second term in Eq. (1)) and of $\mathbf {g}(V_\mathrm{m}, \mathbf {h})$ in Eq. (2) is determined by the respective cell model.

All simulations were performed on two-dimensional rectangular simulation domains, where no-flux boundary conditions were used. In Table 2 the time step *dt*, the diffusion constant *D*, the grid constant *dx*, and the sizes of simulation grids are given for each cell model.

**Table 2:** This table lists the time step *dt*, the diffusion constant *D*, the spatial grid parameter *dx*, the number of grid points along the *x* and *y* axis of the simulation domain $N_x$, $N_y$, respectively, and the resulting system size *A* for each investigated cell model.

Model	*dt* [ms]	*D* [$\hbox {cm}^2/\hbox {s}$]	*dx* [mm]	$N_x \times N_y$	*A* [mm^2^]
AP	0.1	1.5	0.5	$200 \, \times \, 200$	$100 \, \times \, 100$
MS	0.1	0.5	0.8	$150 \, \times \, 150$	$120 \, \times \, 120$
FK	0.1	2.0	1.0	$150 \, \times \, 150$	$150 \, \times \, 150$
BOCF	0.1	2.0	0.8	$576\, \times \, 576$	$460.8 \, \times \, 460.8$

#### Aliev–Panfilov model

The Aliev–Panfilov model^25^ is a two-variable model ($V_\mathrm{m}$ and *v*) with five parameters for cardiac excitation, where the local part of Eq. (1) is given by $\mathbf {f}(V_\mathrm{m}, v) = kV_\mathrm{m} (1-V_\mathrm{m})(V_\mathrm{m}-a)-V_\mathrm{m} v$ and the governing equation of *v* is

3 $$\begin{aligned} \frac{\partial v}{\partial t} = \varepsilon (V_\mathrm{m}, v)(-v-kV_\mathrm{m}(V_\mathrm{m}-a-1)) \, , \end{aligned}$$

with $\varepsilon (V_\mathrm{m}, v) = \varepsilon _0 + \frac{\mu _1 v}{V_\mathrm{m} + \mu _2}$  . The model parameters are given in Table 3.

**Table 3:** This table lists the model parameters used for all simulations of the Aliev–Panfilov model.

Parameter		Parameter	
*a*	0.06	$\mu _1$	0.2
*k*	10	$\mu _2$	0.3
$\epsilon _0$	0.001		

#### Mitchell-Schaeffer model

We used the Mitchell–Schaeffer model^26^ with the adaption from Álvarez et al.^27^, where the local dynamics is described by $\mathbf {f}(V_\mathrm{m}, v) = v(1-V_\mathrm{m})(V_\mathrm{m}-V_\mathrm{m}^\mathrm{rest})^2\frac{1}{\tau _\mathrm{in}} - (V_\mathrm{m}-V_\mathrm{m}^\mathrm{rest})\frac{1}{\tau _\mathrm{out}}$ and

4 $$\begin{aligned} \frac{\partial v}{\partial t} = {\left\{ \begin{array}{ll} \frac{1-v}{\tau _\mathrm{open}} &{} V_\mathrm{m} \le V_\mathrm{m}^\mathrm{crit} \, ,\\ -\frac{v}{\tau _\mathrm{close}} &{} V_\mathrm{m} > V_\mathrm{m}^\mathrm{crit} \, . \end{array}\right. } \end{aligned}$$

The parameters used for the Mitchell–Schaeffer model are shown in Table 4.

**Table 4:** This table lists the model parameters used for all simulations of the Mitchell–Schaeffer model.

Parameter		Parameter	
$\tau _\mathrm{in}$	0.4 ms	$\tau _\mathrm{open}$	130 ms
$\tau _\mathrm{out}$	10 ms	$\tau _\mathrm{close}$	150 ms
$V_\mathrm{m}^\mathrm{crit}$	0.13 a.u.	$V_\mathrm{m}^\mathrm{rest}$	0.04 a.u.

#### Fenton-Karma model

The Fenton-Karma model is a three variable model ($V_\mathrm{m}$, $\mathbf {h}=(v, w)$) with fourteen parameters, where the transmembrane currents $\mathbf {f}(V_\mathrm{m}, \mathbf {h})$ consist of the fast inward current $I_\mathrm{fi}$, the slow outward current $I_\mathrm{so}$, and the slow inward current $I_\mathrm{s{i}}$: $\mathbf {f}(V_\mathrm{m}, \mathbf {h})=\frac{1}{C_\mathrm{m}}\, I_\mathrm{tot}(V_\mathrm{m}, \mathbf {h})=\frac{1}{C_\mathrm{m}}\left( I_\mathrm{fi}(V_\mathrm{m}, \mathbf {h}) + I_\mathrm{so}(V_\mathrm{m}) + I_\mathrm{si}(V_\mathrm{m}, \mathbf {h})\right)$, where $C_\mathrm{m}$ is the membrane capacitance. The exact form of the transmembrane currents and the governing equations of *v* and *w* can be found in Fenton et al.^28^. The model parameters are shown in Table 5.

**Table 5:** The set of parameters used for simulations of the Fenton-Karma model.

Parameter		Parameter		Parameter	
$\mathrm {\tau _{v+}}$	$13.03\,\hbox {ms}$	$\mathrm {\tau _{0}}$	$12.5\,\hbox {ms}$	$\mathrm {\tau _{v1}^-}$	$19.6\,\hbox {ms}$
$\mathrm {\tau _{r}}$	$33.25\,\hbox {ms}$	$\mathrm {\tau _{v2}^-}$	$1250\,\hbox {ms}$	$\mathrm {\tau _{si}}$	$29\,\hbox {ms}$
$\mathrm {\tau _{w}^+}$	$800\,\hbox {ms}$	$\mathrm {u_c}$	0.13 a.u.	$\mathrm {\tau _{w}^-}$	$40\,\hbox {ms}$
$C_\mathrm{m}$	1 a.u.	$\mathrm {\tau _d}$	$0.45\,\hbox {ms}$	$\mathrm {u_v}$	0.04 a.u.
$\mathrm {u_c^{si}}$	0.85 a.u.				

#### Bueno-Orovio-Cherry-Fenton model

The Bueno-Orovio-Cherry-Fenton model^29^ uses four variables ($V_\mathrm{m}$, *v*, *w*, *s*) and three transmembrane currents $I_\mathrm{tot} = I_\mathrm{fi}(V_\mathrm{m}, v) + I_\mathrm{so}(V_\mathrm{m}, v) + I_\mathrm{si}(V_\mathrm{m},w)$. In this study, we use the parameter set denoted as PB defined in Table 1 in the original paper^29^. This parameter set was obtained by fitting the BOCF model such that it reproduces the dynamics of the Priebe-Beuckelmann model^35^.

### Determination of “Dominant Frequency” $f_\mathrm{dom}$

**Figure 6:**
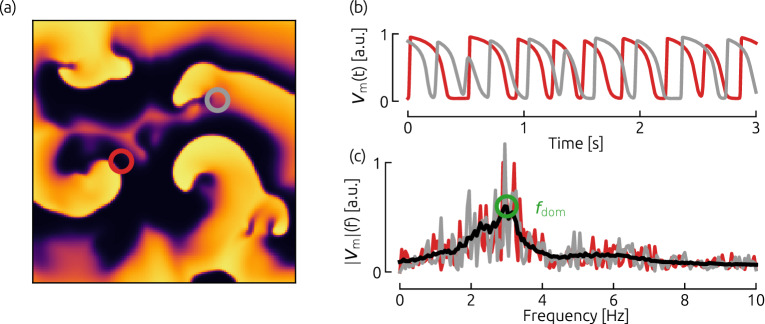
Determination of the dominant frequency $f_\mathrm{dom}$ of the system. The time series of the membrane potential was sampled for each cell model (a representative snapshot is shown in (**a**) for the FK model). The red and gray circles indicate two exemplary locations, where a fraction of the time series is shown in subplot (**b**). Based on these time series, Fourier spectra were computed (shown in subplot (**c**)), which were averaged in the end (black curve in (**c**)). Note, that in practice, this procedure was performed not just for two locations, but for all pixels of the spatial domain. Also, the temporal length of time series which were used for the computation of the Fourier amplitude spectra, was $20\,\hbox {s}$ for all cell models.

In order to characterize the dynamics observed, the “Dominant Frequency” was derived for each cardiac cell model. For this purpose, unperturbed episodes of $20\,\hbox {s}$ of spatio-temporal spiral wave chaos were simulated. Then, Fourier spectra were computed based on time series of the membrane voltage $V_\mathrm{m}$ at each pixel of the simulated domain separately (for an example see Fig. 6). The obtained Fourier amplitude spectra associated with each pixel of the domain were averaged (black line in Fig. 6(c)), and the frequency with the largest amplitude was considered as the dominant frequency $f_\mathrm{dom}$.

### Detection of phase singularities

The number of spiral waves present in the system can be derived by computing the number of phase singularities. For this purpose, in the case of FK simulations we use a two step approach: In a first step, the phase at a specific position on the simulation grid and at a given point in time $\theta (t, x, y)$ is computed, based on the (normalized) membrane potential $V_\mathrm{m}$ and the second dynamical variable *v*:

5 $$\begin{aligned} \theta (t, x, y) = \mathrm {atan2}(V_\mathrm{m}(t, x, y) - V^0_\mathrm{m}, v(t, x, y) - v^0)\, , \end{aligned}$$

where $V^0_\mathrm{m}=0.3$ and $v^0 = 0.1$ are reference values.

In the following, a spiral wave tip is associated with a phase singularity (PS). Phase singularities are determined by performing path integrals along closed paths $\partial \mathcal D$ which enclose small domains of 2$\times$2 pixel of the simulation grid. If the result of such a path integral

6 $$\begin{aligned} \mathrm PS(t, x, y) = \left| \frac{1}{2\,\pi }\oint _{\partial \mathcal{D}}\nabla \theta (t, x, y)\cdot \mathrm {d}\mathbf {l} \right| \end{aligned}$$

is equal to one, a phase singularity and thus a spiral wave tip has been detected at the specific point in time and space.

## Data Availability

The data that support the findings of this study are available from the corresponding author upon reasonable request.
